# Outcome of nonsurgical management of large cyst-like periapical lesions using a modified apical negative pressure irrigation system: a case series study

**DOI:** 10.1186/s12903-024-04110-2

**Published:** 2024-03-15

**Authors:** Danhua Ling, Yun Chen, Gongpei Chen, Yanzhen Zhang, Yanhong Wang, Ying Wang, Fuming He

**Affiliations:** 1 Department of General Dentistry, The Second Affiliated Hospital of Zhejiang University School of Medicine, Hangzhou, Zhejiang Province China; 2 Department of Prosthodontics, Stomatology Hospital, School of Stomatology, Zhejiang University School of Medicine, 166 Qiutao North Road, Shangcheng District, Hangzhou, Zhejiang Province China; 3 School of Stomatology, Zhejiang Chinese Medical University, Hangzhou, Zhejiang Province China; 4 Department of Comprehensive Dentistry, Jiangnan Hospital Affiliated to Zhejiang Chinese Medical University, Hangzhou, Zhejiang Province 311221 China

**Keywords:** Apical negative pressure irrigation, Nonsurgical root canal therapy, Large cyst-like periapical lesions, Volumetric change

## Abstract

**Objective:**

To assess the effectiveness of a self-constructed modified apical negative pressure irrigation (ANPI) system employing commonly used clinical instruments in nonsurgical root canal therapy (NSRCT) for large cyst-like periapical lesions (LCPLs).

**Methods:**

From 2017 to 2022, 35 patients diagnosed with LCPLs (5-15 mm) via preoperative clinical and radiographic evaluations of endodontic origin underwent NSRCT combined with ANPI. These patients were subjected to postoperative clinical and radiographic follow-up at 3 months, 6 months, 1 year, 2 years, 3 years, and 4 years, with a CBCT scan specifically conducted at 6-month follow-up. Through the reconstruction of three-dimensional cone beam computed tomography (CBCT) data, an early prognosis was facilitated by monitoring changes in lesion volume. Various treatment predictors—including sex, type of treatment, lesion size, preoperative pain, jaw, type of teeth involved, sealer extrusion, and the number of root canals—were meticulously analyzed. The evaluation of post-treatment outcomes leveraged both clinical observations and radiographic data collected during the follow-up periods. The Kruskal‒Wallis test and one-way ANOVA were also conducted to determine the independent factors influencing treatment outcomes. A significance level of 5% was established.

**Results:**

Thirty-five teeth from 35 patients with a median age of 28 years (range 24–34) were treated; the median follow-up duration was 19 months (range 12–26). The overall success rate was 91.4%, with a median lesion reduction of 77.0% (range 54.2–96.4%) at 6 months. Patients under 30 years of age exhibited a significantly greater success rate than older patients did (100.0% vs. 80.0%, *p* = 0.037). Other factors, such as sex, jaw, treatment type, preoperative pain, cyst size, tooth location, sealer extrusion, and the number of roots, did not significantly impact treatment outcomes.

**Conclusions:**

Despite limitations related to the observational case-series study design and relatively small sample size, our findings suggest that utilizing the ANPI in the NSRCT for LCPLs may hold promise. The notably higher success rate in patients younger than 30 years is worth noting.

## Clinical significance

The modified ANPI system offers a viable treatment modality for managing outcomes in patients with LCPLs.

## Strengths and limitations of this study

- This is the first study to explore the outcomes of apical negative pressure irrigation in nonsurgical root canal therapy for large cyst-like periapical lesions.
- A modified apical negative pressure irrigation system is designed to overcome limitations such as high costs, complex operation, the risk of injury due to improper usage, and limited availability in certain dental practices.
- The imaging examinations in this study were consistently scheduled for the 6-month follow-up period, which is more significant than what has been observed in other studies with inconsistent follow-up times.
- The limitations of this study include its retrospective case series design and the limited number of included patients.

## Introduction

Large cyst-like periapical lesions (LCPLs) originate from the epithelial remnants of Malassez located within the periodontal ligament [1]. These lesions manifest as pathological cavities fully lined with nonkeratinized stratified squamous epithelium of varying thickness, forming a three-dimensional structure within apical periodontitis [1]. It is important to emphasize that LCPLs should be regarded as indicators of cyst formation due to apical periodontitis [2, 3]. The prevalence of LCPLs varies between 6% and 55% [4–6]. These defects more frequently manifest in the maxilla than in the mandible, with the maxillary lateral and central incisors being the most commonly affected teeth. While benign in nature, LCPLs induce inflammation and bone destruction, necessitating prompt treatment.

The treatment approach for LCPLs depends on the severity of the disease and the patient’s medical history. Nonsurgical treatment refers to nonsurgical root canal therapy, while surgical alternatives include endodontic surgery and tooth extraction. NSRCT represents a highly predictable and reliable treatment for LCPLs with a commendable success rate ranging from approximately 82.2–83.3% [3, 6]. Research has indicated that the survival rates of patients after NSRCT and apical surgery are not significantly different following the failure of primary root canal therapy [2]. Moreover, researchers have unveiled the utility of NSRCT in managing LCPLs [7, 8].

Histological investigations have demonstrated the role of vascular endothelial growth factor (VEGF) in promoting cellular chemotaxis and plasma protein extravasation, culminating in fluid accumulation within LCPLs [9]. This, in turn, results in increased hydrostatic pressure, thereby contributing to lesion expansion. The accumulation of metabolic byproducts establishes a notable osmotic gradient, driving fluid flow into the cystic cavity and fostering lesion wall expansion [9, 10]. NSRCT mitigates hydrostatic pressure through aspiration and decompression, potentially expediting the healing process of LCPLs [11].

Syringe irrigation is commonly employed for root canal irrigation. However, multiple studies have demonstrated that positive pressure irrigants delivered via syringes and needles cannot adequately address root canal irregularities, lateral canals, or the isthmi [12, 13]. Furthermore, irrigant and debris may be extruded from the apical foramen, potentially damaging apical tissue. The concept of apical negative pressure irrigation (ANPI) was first introduced by Fukumoto et al. [14]. ANPI entails applying negative pressure to the root canal, creating a vacuum effect that facilitates the removal of bacterial and dentinal debris. This is achieved by inserting a needle into the root apex and using an instrument to apply negative pressure and effective decompression while introducing an irrigant solution to flush out bacterial and dentinal debris. The vacuum effect ensures thorough cleaning and disinfection of the root canal, thus yielding predictable treatment outcomes [15]. Other studies have shown that ANPI prevents apical extrusion of irrigants and is particularly effective for narrow isthmi roots [16–18]. Fluid dynamics research has shown that ANPI microcapsules can create fluid flow in the apical region and establish negative apical pressure values for effective decompression of LCPL [13]. This finding suggested that ANPI may enhance the decompression effect inside LCPLs during the NSRCT process. Researchers believe that successful resolution of a large periapical lesion can be achieved through accurate diagnosis and the implementation of an appropriate treatment strategy, thereby eliminating the need for surgical intervention. ANPI may offer advantages for intra-LCPL decompression of exudates [19]. Despite these advantages, the effectiveness of the ANPI remains a subject of debate. Several popular endodontic irrigation systems, including EndoVac, EasyClean, and VATEA, have limitations, such as high costs, complex operation, risk of injury due to improper usage, and limited availability in certain dental practices [20]. Thus, our aim was to address these issues by developing a modified ANPI system using readily available clinical instruments, offering advantages such as affordability, ease of use, and broad applicability [21].

While NSRCT has been widely employed as the primary treatment for LCPLs, efforts should be made to enhance its success rate and expedite the treatment process. To the best of our knowledge, no previous study has explored the outcomes of the ANPI in NSRCT for LCPLs. Therefore, this case series study was undertaken to assess the efficacy of the ANPI in treating LCPLs in NSRCT using a modified ANPI model.

## Materials and methods

### Patient selection

No previous study has explored the outcomes of the ANPI in patients with LCPLs in the NSRCT. Therefore, before commencing our study, we analyzed the results of normal root canal treatment performed by our odontist combined with the ANPI (*n* = 50). A success rate of 92% was achieved. Considering the 10% relative precision and 95% confidence interval, a minimum of 29 teeth needed to be included. Considering a 20% attrition rate, a sample of 35 patients (35 teeth) with LCPLs was included [3]. All patients underwent NSRCT for LCPLs at the Department of General Dentistry, The Second Affiliated Hospital of Zhejiang University School of Medicine, from January 2017 to June 2022. The diagnosis was based on preoperative clinical and radiographic criteria outlined in a prior study [4, 22]. These criteria included the presence of a non-vital tooth, a radiolucent area ≥ 5 mm with a distinct radiopaque border affecting one or more teeth, and confirmation that the lesion originated from the tooth. This research was approved by the ethics committee of the Second Affiliated Hospital of Zhejiang University School of Medicine (No: 2022–0709 (I2022907)).

Patients aged 12–60 years with complete root development; no systemic diseases (e.g., uncontrolled hypertension, diabetes, AIDS); complete preoperative, postoperative, and follow-up CBCT data; and a recall period of at least 6 months were included. Additionally, the probing depth of the teeth was required to be ≤ 4 mm for optimal periodontal health, and the maximum diameter of the periapical lesion, according to the radiographic data, was between 5 and 15 mm, with the only drainage pathway being through the root canal, keeping the adjacent bony structure intact.

Patients with poor long-term tooth prognoses, such as root canal imperforation, periodontal disease, cracks extending into the deep pulp chamber, or vertical root fractures, were excluded.

### Model construction

**Fig. 1:**
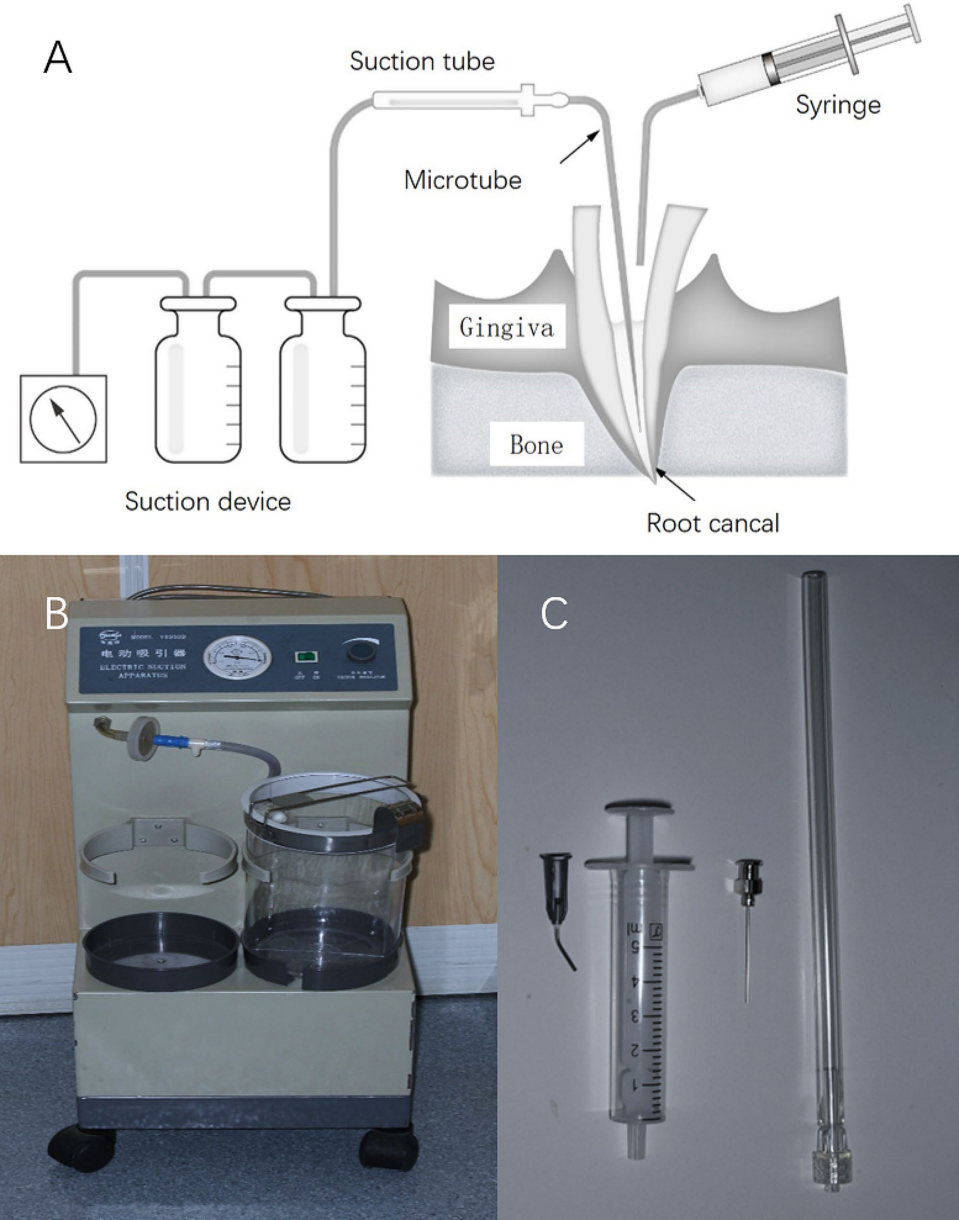
Structure of a modified ANPI system. (**A**) Model structure depiction. (**B**) Schematic of the suction device. (**C**) A specialized metal needle tip was used as the microtube for extraction of irrigation solution from the apical region

A modified ANPI system was created based on previous literature and ANPI principles [14, 21]. The system included a suction device (Simanfeng, Shanghai, China), a specially designed disposable suction tube (Mete Medical, Shanghai, China), a specialized metal needle tip (Zhejiang Guangci, Zhejiang, China), and a disposable 5 mL syringe (Cofoe, Shanghai, China). The structure of the model is depicted in Fig. 1A, and the structure of the suction device is shown in Fig. 1B. The specialized metal needle tip was initially employed as the microtube for irrigation solution extraction from the apical region, with the disposable syringe ensuring a consistent flow rate to the root canal (Fig. 1C). The microtube needle tip was available at three diameters (0.3 mm, 0.5 mm, and 0.7 mm), allowing for selection based on the root canal’s diameter for improved adaptability. The suction device, which functions as a negative pressure aspirator, is linked to a suction tube, which is further connected to a specialized metal needle tip. In the process of negative pressure irrigation, a 5 mL syringe served to deliver exogenous irrigation fluid to the upper region of the root canal. Concurrently, the metal needle tip was positioned deep within the root apex. The negative pressure created by the suction device relies on the metal needle tip, facilitating the aspiration of the irrigation fluid into the suction device. This combination of components ensures the efficient removal of irrigation fluid from the root apex.

### Treatment protocol

All procedures were performed by a single operator. NSRCT was conducted using the modified ANPI system. Informed consent was obtained from patients after discussing the treatment plan and long-term prognosis. A rubber dam was used for a dry field, and access to the root canal was established. An electronic apex locator (Dentsply Maillefer, Ballaigues, Switzerland) and a #8 K-file (Mani, Tochigi, Japan) were used to determine working lengths. Root canal preparation was carried out until the canal reached a #15 size, with each K-file advanced 2 mm above the apical foramen.

**Fig. 2:**
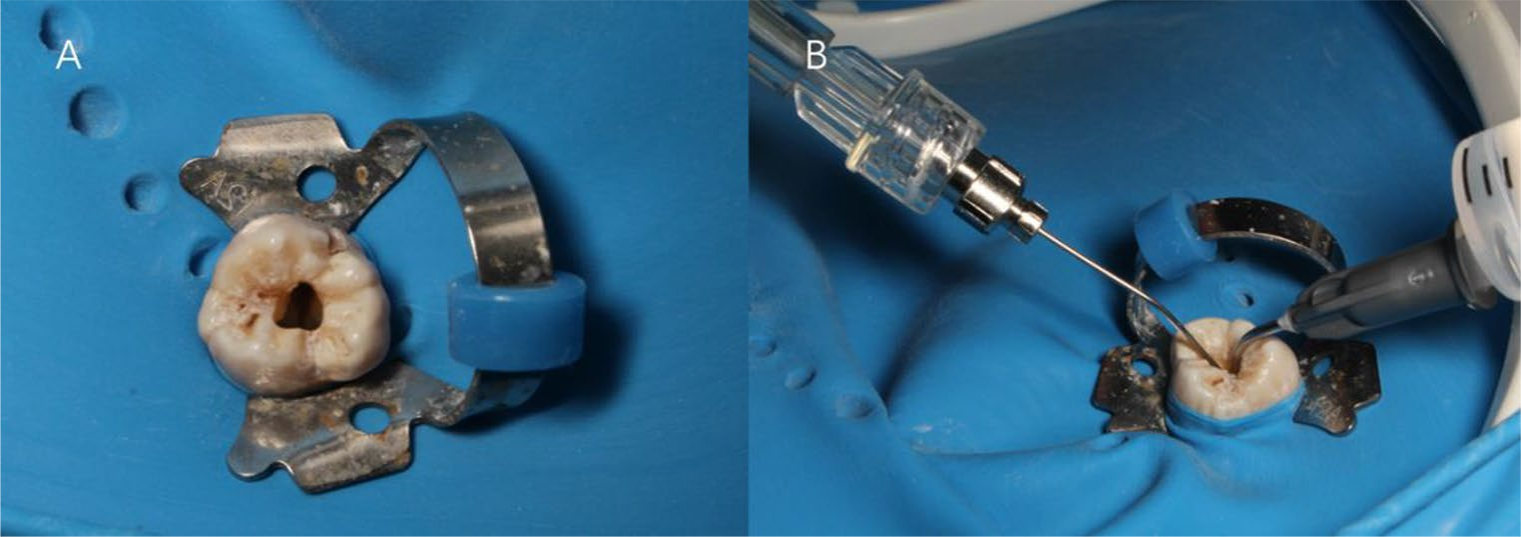
(**A**) The view of the exposed maxillary molar pulp chamber before ANPI. (**B**) ANPI during the irrigation process. The microtube on the left was used to extract the irrigation solution (left). A 5 mL syringe serves to deliver exogenous irrigation fluid to the upper region of the root canal (right)

The ANPI system was employed for cystic fluid drainage. In this phase, a microtube with a diameter of 0.5 or 0.7 mm was utilized to facilitate optimal access to the apical area while simultaneously preventing canal blockage by debris. Root canal preparation was carried out using Ni‒Ti rotary instruments (Dentsply Maillefer, Ballaigues, Switzerland) following the crown-down preparation method. The canals were irrigated with a combination of 1% sodium hypochlorite and 0.9% normal saline using the ANPI system. This entire process of instrumenting and enlarging the root canals aimed to ensure that all canals reached size F3. Notably, particular attention was given to narrow or curved canals, which were prepared up to size F2. The needle tip diameter was chosen meticulously to ensure access within 2 mm of the apical foramen. The flow rate for each irrigation cycle was 5 mL/min, and the volume of the individual solution was 5 ml. Additionally, the suction device maintained a negative pressure value of 0.05 MPa. Following the use of different Ni‒Ti rotary instruments, the root canal was irrigated with the ANPI system (Fig. 2). During irrigation, the microtube needle tip was precisely positioned within 2 mm of the apical foramen. Subsequently, the canals were dried using paper points, and a calcium hydroxide [Ca(OH)_2_] paste (Gapadent, Tianjin, China) was applied. One week after the procedure, the root canal was irrigated again with the ANPI system. This procedure was repeated 3 times until the affected tooth no longer showed significant inflammatory exudate or pain. Otherwise, the affected tooth can only receive apical surgery. The canals were filled with gutta-percha (Gapadent, Tianjin, China) and iRoot SP (Innovative BioCeramix, Vancouver, BC, Canada) using the single cone technique. In addition, the access cavity was sealed with glass ionomer cement (GC-Fuji IX Japan). A final digital periapical radiograph (PA) was obtained to confirm proper obturation. Upon completion of the root canal treatment, patients were referred to their general dentists for the placement of a permanent coronal restoration. If requested by the referring dentist, a permanent restoration was placed before the final restoration.

Patients were scheduled for follow-up appointments at intervals of 3 months, 6 months, 1 year, 2 years, 3 years, and 4 years. Preoperative and 6-month postoperative CBCT scans were performed using Planmeca Romexis Viewer 4.5.0R (Planmeca Oy, Helsinki, Finland). Subsequently, 3D Slicer 5.0.3 software was used to create a 3D radicular lesion model from the exported 3D CBCT data. This approach facilitated the calculation of the lesion volume. PAs were taken during recall visits, excluding the 6-month appointment, to monitor the healing status of large cyst-like periapical lesions.

### Clinical and radiographic evaluation

Clinical and radiographic evaluations were also conducted by another operator not involved in the previous procedures. The clinical evaluation assessed symptoms such as the presence of a sinus tract, sensitivity to percussion and palpation, swelling, the presence of periodontal pockets, and pain.

**Fig. 3:**
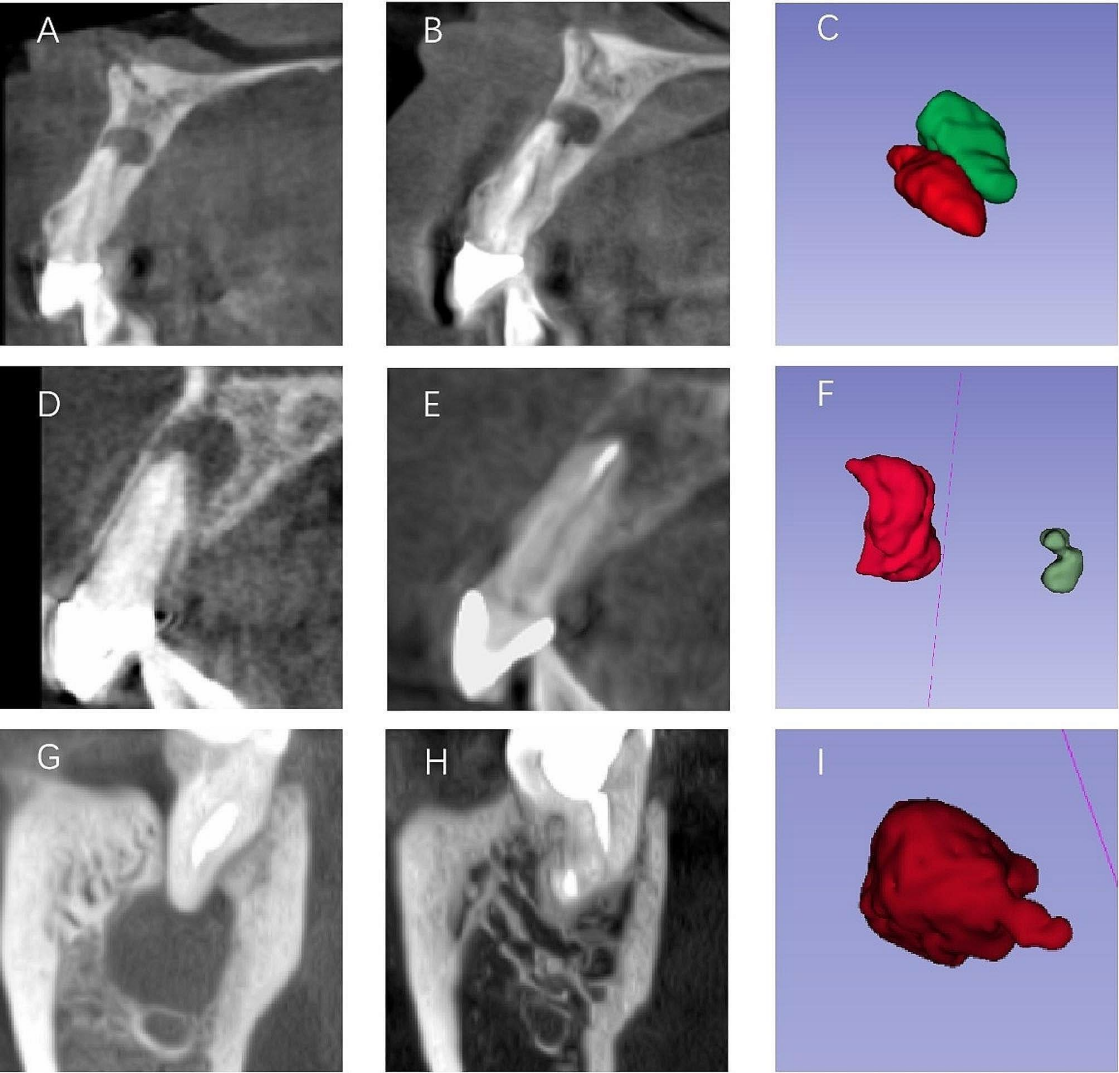
Cases illustrating each healing category. (**A**, **D**, and **G**) Sagittal-sectional preoperative CBCT, (**B**, E, and **H**) sagittal-sectional postoperative CBCT, and (**C**, **F**, and **I**) 3D reconstruction images obtained at the 6-month recall. The red models represent preoperative lesions, while the green models depict postoperative lesions. (**A**–**C**) Treatment was considered a “failure” because the lesion volume did not decrease. (**D**–**F**) The lesion was reduced in size at the 6-month recall, so this case was considered a ‘‘success”. (**G**–**I**) The maximum diameter of this large periapical lesion was 11 mm. The lesion was undetected at the 6-month recall, so this case was also considered a ‘‘success”

CBCT images at the 6-month follow-up were assessed blindly twice by a third operator with a 2-week interval between the two assessments [23]. The teeth were classified according to the following criteria: patients with both healed and healing outcomes were considered successful, while non-healed patients were considered failures (Table 1). Figure 3A–I shows the cases in each healing category.

**Table 1:** Classification system for teeth after treatment

Classification	Clinical evaluation	Radiographic evaluation	Outcome
Healed	Asymptomatic teeth	Lesion undetected,	Success
Healing	Asymptomatic teeth	Lesion reduced in size more than 20%	Success
Nonhealed	Nonfunctional, symptomatic teeth	Lesion reduced in size less than 20%, or enlarged radiographic	Failure

The calculation of the lesion’s volume was conducted using 3D Slicer 5.0.3 software at the highest possible resolution (0.2 mm for both slice thickness and interval), employing semi-automatic active contour segmentation. The segmentation process utilized the “fast marching” method, as described by Pichon et al. [24], facilitating a semiautomatic approach. This software enables straightforward quantification of defect volume in cubic millimeters (mm^3^). The criterion for the absence of a periapical lesion was a radiographic periodontal ligament space less than double its width, following the guidelines of Schloss et al. [25]. The percentage reduction in the lesion volume was determined using the following formula: percentage change in lesion volume (%) = ((preoperative volume - postoperative volume)/preoperative volume) × 100. In multirooted teeth, when the range of the LCPL encompasses all the roots of the affected tooth simultaneously, its assessment and comparison align with those of a single-rooted tooth treated with the NSRST. However, if the LCPL does not cover all the roots of the affected tooth or if the tooth’s roots receive several LCPLs independently, the evaluation and comparison of the tooth’s outcome are determined by the root categorized under the ‘worst’ diagnostic outcome [26, 27].

The success of treatment hinges on thorough clinical evaluations and radiographic imaging results. At the 6-month follow-up, the determination of early treatment success relies on both clinical evaluations and lesion volume changes. For follow-up appointments at 1 year or beyond, treatment efficacy can be assessed by integrating clinical evaluations with intraoral PA imaging.

### Statistical analysis

The statistical analysis was performed using SPSS v23.0 software (IBM Corp, Armonk, NY). The intraobserver agreement for the radiographic assessments was assessed by the intraclass correlation coefficient. The data are presented as the median (interquartile range [IQR]) because of a nonnormal distribution. The data were analyzed using SPSS or GraphPad Prism (GraphPad Software, California, USA). The mean values of the two groups, such as treatment types, were compared using the Kruskal‒Wallis test followed by Dunn’s post hoc test. The mean values of three groups, such as types of teeth, were compared using one-way ANOVA followed by Tukey’s post hoc test. The chi-square test was used for categorical data analysis. A *p*-value < 0.05 was considered to indicate statistical significance.

## Results

A total of 35 teeth from 35 patients, with a median age of 28 (24–34) years, received treatment from our endodontist (Table 2). The median follow-up time was 19 (12–26) months. One tooth was extracted due to significant pain at the 6-month follow-up. The other two teeth required periapical surgery because of increased volume and clinical discomfort. The overall success rate was 91.4%. An intraclass correlation coefficient (ICC) of 0.94 demonstrated a strong level of consistency in repeated data measurements. Given the high ICC values, the average of the two measurements was used for volume assessment. The median preoperative volume was 416.5 (252.5–782) mm^3^. The median postoperative volume was 90 (12–190) mm^3^. The postoperative volume of the LCPL was significantly smaller than the preoperative volume (*p* < 0.05). The median percentage of lesion reduction was 77.0 (54.2–96.4)% at 6 months. Patients under 30 years of age had a significantly greater success rate than older patients (100.0% vs. 80.0%, *p* = 0.037). None of the other factors, including sex, jaw, lesion size, tooth location, sealer extrusion, or the number of roots, significantly influenced the success rate of the treatment (Table 3). The other study parameters did not significantly impact the lesion’s volume reduction rate.

**Table 2:** Demographic data distribution

Variables	Result
Patients (n)	35
Men	16
Women	19
Age, Median (IQR)	28 (24–34)
Jaw (n)	
Mandibular	13
Maxillary	22
Incisor	18
Molars and premolars	17

*Abbreviations*: IQR, Interquartile range

**Table 3:** Distribution of the treatment outcomes by study factors and demographics

		Success, n (%)	Preoperative size, Median (IQR) (mm^3^)	Postoperative size, Median (IQR) (mm^3^)	Reduction percentage, Median (IQR) (%)
Gender					
	Male (16)	15 (93.8)	425.8 (284.6–768.6)	61.5 (9.4–182.5)	81.5(62.3–97.7)
	Female (19)	17 (89.5)	338.5 (217.5–889.5)	93 (12–327)	71.7(52.1–95.9)
P		0.65	0.53	0.55	0.35
Age (year)					
	≤ 30 (20)	20 (100)	425.8 (262.1–814.3)	137.5(13.5–183.8)	76.9(60.1–94.4)
	> 30 (15)	12 (80)	297(251–708)	31(12–216)	86(27.3–96.4)
P		0.037*	0.57	0.69	0.92
Treatment type					
	Initial (21)	20 (95.2)	338.5(254.3–743.3)	59(8.5–162.5)	81.5(61.6–97.7)
	Retreatment (14)	12 (85.7)	512(215.5–825)	122.5(176.5–354.3)	60.9(46.5–96.1)
P		0.39	0.81	0.24	0.13
Size (mm)					
	> 10 (14)	12 (85.7)	857.3(676–988.1)	155(20–360)	80.3(57.9–96.4)
	≤ 10 (21)	20 (95.2)	274(216.5–372.3)	36(10–150)	70.3(53.7–96.9)
P		0.30	< 0.001*	0.080	0.89
Pain before surgery					
	Yes (22)	20 (90.9)	420.5(269.5–891)	51(2.63–224.3)	85.8(60.8–99.7)
	No (13)	12 (92.3)	416.5(173.8–718.3)	135(23–190.5)	70(28.4–81.5)
U/χ^2^ (p)		0.89	0.20	0.47	0.070
Jaw					
	Maxillary (22)	20 (90.9)	522(278.9–889.6)	91.5(12.4–304.5)	80.2(57.9–96.7)
	mandibular (13)	12 (92.3)	294(142.3–601.5)	59(7.8–162.5)	71.7(53.7–93.7)
P		0.89	0.082	0.56	0.78
Type of teeth					
	Anterior (18)	17 (94.4)	377.5(246.3–764)	74.5(8.8–177.8)	77.9(53.5–97.5)
	Premolar (9)	8 (88.9)	274(141.3–367.3)	31(12–112)	85.6(58.7–97.9)
	Molar (8)	7 (87.5)	857(548.3–893)	182.5(143–409)	65.7(52.4–84.9)
P		0.80	0.008*	0.074	0.58
Sealer extrusion					
	Yes (4)	4 (100)	803.5(420.1–882.4)	97.5(10.8–260.8)	83.6(66.9–96.4)
	No (31)	29 (93.5)	406(251–728.5)	90(12–190)	76.8(53.2–96.4)
P		0.52	0.12	0.88	0.41
Number of root canals					
	1(25)	23 (92)	297(224.8–654,3)	31(8.5–162.5)	79(57.7–97.7)
	2(1)	1 (100)	256	59	76.9
	3(9)	8 (88.9)	825(450.5–892)	175(91.5–392)	71.7(52.7–85.8)
P		0.92	0.042*	0.13	0.71

The data are presented as medians (interquartile ranges [IQRs]). The mean values of the two groups, such as treatment types, were compared using the Kruskal‒Wallis test followed by Dunn’s post hoc test. The mean values of three groups, such as types of teeth, were compared using one-way ANOVA followed by Tukey’s post hoc test

*, *p* < 0.05

## Discussion

The aspiration and decompression of LCPLs play crucial roles in reducing osteoclastic activity, preventing further damage to the surrounding bone and tissues. Instrumentation beyond the apex during NSRCT can alleviate hydrostatic pressure in the LCPL, facilitating adequate drainage of cystic fluid and lesion healing [6]. ANPI is a promising technique for root canal disinfection and biofilm removal from artificial canals. Several studies have demonstrated its effectiveness in removing the smear layer, flushing out debris and irrigant from the root canal, and preventing apical extrusion of irrigant, thereby enhancing the success of root canal treatment [16, 17, 28]. In contrast to traditional positive pressure irrigation, which pushes irrigating solutions into the canal, ANPI creates a suction effect. This suction ensures that the irrigating solution reaches the entire length of the canal, including the apical third, without forcing fluids or debris beyond the root apex into the periapical tissues. ANPI leverages negative pressure to enhance the removal of infected material, pus, and debris from within the lesion, promoting a more effective healing process by alleviating the build-up of pressure and facilitating the clearance of pathological contents [19, 28]. Fluid dynamics research has shown that ANPI microcapsules can create fluid flow in the ramification and establish negative apical pressure values for effective decompression of the LCPL [13]. However, it is important to note that ANPI may not be available in all dental practices, limiting patient access to this treatment option. In our study, we achieved positive results by using simple instruments based on the principles of the ANPI for clinical application. The advantages of our method include its simplicity, cost-effectiveness, and ease of use in clinical practice. The study’s flow rate was based on commercially available ANPI devices [16, 20]. However, this modified system still requires further refinement to optimize its clinical utility.

The reported success rates of NSRCTs in many studies vary between 70.9% and 90.9% [3, 29, 30]. These discrepancies can be attributed to differences in techniques, criteria for treatment success, length of follow-up time, and other factors. Success rates are influenced by various anatomical factors and treatment methods. One common reason for NSRCT failure is perceived leakage around the canal filling material and missing canals [31]. The use of a microscope can aid in locating missing canals and visualizing root obstructions [32, 33]. The incorporation of ultrasonic instruments into endodontic procedures has significantly improved the removal of canal obstructions and the effectiveness of irrigation [34]. Teeth without periapical radiolucency tend to exhibit better outcomes [6, 35]. In our study, the overall success rate was 91.4%, which is higher than that of previously reported results. This finding suggested that the presence of LCPLs does not necessarily impact a tooth’s prognosis when combined with the ANPI. It is worth noting that our study considered teeth as the study unit, whereas another study used roots as units [36]. When considering tooth roots as study units, the success rate was 90.74%, which aligns with the existing results. This could be attributed to the large sample sizes in our study, which involved multiple roots of teeth. A recent prospective cohort study using CBCT imaging reported a success rate of 82.2%, which is lower than our findings. This discrepancy may be due to the aspiration-irrigation technique used in the previous study. This technique carries the potential risk of mucosal inflammation, persistent surgical site complications, and potential lesion reinfection [37]. In contrast, the ANPI procedure used in our study effectively decompressed lesions and mitigated the risk of infection. Furthermore, the presence of a preoperative palatal cortical bone defect may have influenced osseous healing. The periosteum plays a critical role in endogenous bone repair and remodeling, acting as a reservoir of osteo-competent periosteum-derived progenitor cells [38]. Damage to the periosteum and erosion of the cortical bone could lead to delayed healing. Unlike previous studies, we excluded patients with cortical bone defects during patient selection, enhancing the reliability of the results and resulting in a greater success rate than in conventional studies.

Both healed and healing patients were considered successful outcomes, while non-healed patients were considered failures [29]. Our study differs from previous research in that we utilized CBCT scans to measure volumetric changes in LCPLs at the 6-month recall, enabling us to evaluate early treatment success. CBCT imaging is significantly more accurate at detecting the size, shape, and location of periapical lesions than is PA imaging. However, PA radiographs have limitations in consistently revealing the true nature and location of periradicular disease, especially when a bone lesion is within the cancellous bone and the overlying cortical bone is substantial [23]. Moreover, 30–45% of periapical lesions are missed in PA radiographs [39]. In one study in which lesion volumes were measured before and after root canal treatment, CBCT and PA imaging, respectively, showed successful outcomes for 55 (77.5%) and 63 (88.7%) roots [23]. Another recent study reported that when retreatment outcomes were assessed using PA radiographs, the success rate was 93%, but in CBCT assessments, the success rate was lower, at 77% [40]. These findings suggest that CBCT may be a more reliable tool for assessing treatment outcomes. In our study, the postoperative volume of LCPLs was significantly smaller than the preoperative volume (*p* < 0.05). The mean percentage of lesion reduction was 77.0 (54.2–96.4)% at the 6-month follow-up. The observed volume change in our study aligns with previous findings reporting changes of 75% or 76.8% [3, 23]. The distinction lies in the timing of CBCT imaging. In our study, CBCT imaging was performed at the 6-month post-surgery mark, while a previous study conducted imaging between 10 and 37 months after the procedure [23]. The relatively short healing period during CBCT imaging in our study suggested that the lesion is likely to continue healing, resulting in a further decrease in volume over time. We anticipate that this study will yield even more promising results. Additionally, the consistent follow-up period for CBCT imaging enhances the scientific validity of this research.

We investigated various patient and treatment factors, such as sex, age, jaw, type, retreatment, preoperative pain, lesion size, and number of roots, as well as treatment factors such as sealer extrusion. While several factors were associated with nearly a 15% difference in the success rate and the percentage of volumetric decrease, these variations were not statistically significant, except for patient age. Studies have suggested that tooth location (jaw) does not significantly affect the survival rate of NSRCT [3, 41]. Therefore, long-term NSRCT outcomes were not influenced by tooth location or type, which is consistent with our results. However, a previous study revealed that the frequency of potential failures varied based on the tooth’s location [31]. Leaky canals were the primary cause of anterior tooth failure, while missing canals were the primary cause of posterior tooth failure. The complex anatomy of molars makes access and visibility difficult, potentially increasing the likelihood of missing a canal, such as the second mesiobuccal canal of the maxillary molar [42]. Although it remains unclear whether tooth type and position affect NSRCT prognosis, it is evident that different teeth require different treatment interventions.

Some studies suggest that older patients may have worse prognoses due to factors such as decreased bone density and compromised immune systems. Older patients may have a higher risk of complications during surgical or nonsurgical treatments [43, 44]. In our study, patients under 30 years of age had a significantly greater rate of NSRCT success, which is in line with the findings of several previous studies [44]. However, we could not draw similar conclusions regarding volumetric changes, possibly due to our small sample size. Biological differences between sexes could impact the healing process or response to treatment due to differences in hormonal levels, immune response, or bone density [45]. However, some clinical studies have shown that sex does not significantly affect the outcome of treatment for LCPLs [3, 46]. In this study, sex did not influence treatment outcomes, which is consistent with previous findings.

Retreatment poses several challenges, such as removing the previous obturation material, correcting procedural errors from the initial treatment, locating missed canals, and eliminating potentially therapy-resistant bacteria. As a result of these challenges, the prognosis for patients receiving retreatment of normal teeth is generally less favorable than that for patients receiving initial treatment [33]. Nonetheless, our findings do not reveal this discrepancy, potentially due to the superior flushing efficiency and the lesion cavity decompression achieved through the ANPI within the root canal.

The presence of preoperative pain is an important factor that influences the severity of the disease, diagnostic considerations, and treatment decision-making in clinical practice. However, neither current experimental results nor extensive follow-up studies have shown that preoperative pain significantly affects the outcome of endodontic treatment [29, 33, 47]. This is destined to be a challenging issue, as pain is a broad and multifaceted concept with variations in type, severity, and location. Therefore, research on pain may be conducted with a more nuanced classification system to yield meaningful results.

Sealer extrusion is a common occurrence in root canal treatment [29]. In the present study, iRoot SP, known for being a bioceramic-based root canal sealer with outstanding biocompatibility and bioactivity, was utilized. According to our results, sealer extrusion did not affect the treatment outcome. The reason for this difference may be that iRoot SPs can generate hydroxyapatite in the presence of tissue fluid, thereby promoting the regeneration of periapical tissues. Moreover, it has antimicrobial properties, effectively preventing bacterial colonization within the root canal system [48, 49]. The extrusion of biocompatible root canal filling materials does not impede the healing process of periapical tissues.

However, few studies have directly examined the impact of the number of dental roots on endodontic therapy efficacy [47]. Conversely, researchers frequently examine various tooth positions and types. Whether the number of dental roots influences the outcome of nonsurgical treatment for LCPLs may vary depending on several factors, including the size and location of the lesion, the specific treatment approach, and the inherent anatomical complexities associated with multirooted teeth. However, the findings of this study did not yield a positive conclusion, aligning with previous research results [47].

A previous study indicated that the presence of preoperative radiolucency was not a significant predictor of NSRCT success but suggested that larger lesions may require longer follow-up periods [35]. Another study revealed that nonsurgical management can effectively control the progression of LCPLs (> 10 mm) but revealed no preoperative factors related to treatment failure [3]. In this study, we also investigated the efficacy of NSRCT for lesions with a diameter > 10 mm, and our results were 85.71%. This result is larger than that of a previous study (82.2%). Although the success rate of NSRCT with a diameter < 10 mm was nearly 10% greater than that of other stents, no significant difference was observed. One study revealed that treating lesions with a diameter < 5 mm had significantly greater success than treating lesions with a diameter > 5 mm [29, 50]. This may be due to the decreased availability of osteoblast progenitors in larger lesions and the increased likelihood of such lesions being cystic [50]. Given the retrospective design and limited sample size of the study, it is challenging to establish whether lesion size significantly affects NSRCT efficacy.

## Conclusion

Despite the limitations of being an observational case-series study with a small sample size, our positive results obtained using the ANPI in the NSRCT suggest that it may be a promising approach for treating LCPLs. Furthermore, our findings indicate that patients under 30 years of age may have a higher success rate with NSRCTs. These results can guide the design of future clinical trials with more precise power analyses to further investigate the efficacy of this technique.

## Data Availability

The original contributions presented in the study are included in the article, further inquiries can be directed to the corresponding author.

## Abbreviations

ANPI: Apical negative pressure irrigation
NSRCT: Nonsurgical root canal therapy
LCPLs: Large cyst-like periapical lesions
CBCT: Cone-beam computed tomography
VEGF: Vascular endothelial growth factor
